# Missing effects of zinc in a porcine model of recurrent endotoxemia

**DOI:** 10.1186/1471-2482-5-22

**Published:** 2005-10-20

**Authors:** Carsten J Krones, Bernd Klosterhalfen, Michael Anurov, Michael Stumpf, Uwe Klinge, Alexander P Oettinger, Volker Schumpelick

**Affiliations:** 1 Department of Surgery, Technical University of Aachen, Pauwelsstr. 30, 52074 Aachen, Germany; 2 Institute of Pathology, Technical University of Aachen, Pauwelsstr. 30, 52074 Aachen, Germany; 3 Joint Institute for Surgical Research, Leninskie Gory, Moscow 119992, Russian Federation

## Abstract

**Background:**

Chronic human sepsis often is characterised by the compensatory anti-inflammatory response syndrome (CARS). During CARS, anti-inflammatory cytokines depress the inflammatory response leading to secondary and opportunistic infections. Proved *in vitro *as well as *in vivo*, zinc's pro-inflammatory effect might overcome this depression.

**Methods:**

We used the model of porcine LPS-induced endotoxemia established by Klosterhalfen et al. 10 pigs were divided into two groups (n = 5). Endotoxemia was induced by recurrent intravenous LPS-application (1.0 μg/kg E. coli WO 111:B4) at hours 0, 5, and 12. At hour 10, each group received an intravenous treatment (group I = saline, group II = 5.0 mg/kg elementary zinc). Monitoring included hemodynamics, blood gas analysis, and the thermal dilution technique for the measurement of extravascular lung water and intrapulmonary shunt. Plasma concentrations of IL-6 and TNF-alpha were measured by ELISA. Morphology included weight of the lungs, width of the alveolar septae, and rate of paracentral liver necrosis.

**Results:**

Zinc's application only trended to partly improve the pulmonary function. Compared to saline, significant differences were very rare. IL-6 and TNF-alpha were predominately measured higher in the zinc group. Again, significance was only reached sporadically. Hemodynamics and morphology revealed no significant differences at all.

**Conclusion:**

The application of zinc in this model of recurrent endotoxemia is feasible and without harmful effects. However, a protection or restoration of clinical relevance is not evident in our setting. The pulmonary function just trends to improve, cytokine liberation is only partly activated, hemodynamics and morphology were not influenced. Further pre-clinical studies have to define zinc's role as a therapeutic tool during CARS.

## Background

Although most patients survive the initial insult of a hyper-inflammatory sepsis syndrome, they remain at an increased risk of secondary or opportunistic infections. Basic course is the compensatory anti-inflammatory response syndrome (CARS) which derives from sustained or chronic stages of sepsis [1,2]. During CARS, anti-inflammatory members of the cytokine network as IL-10, TGF-β, and IL-1RA depress the inflammatory response, thus reversing the effects of pro-inflammatory cytokines as TNFα, IL-1, and IL-6 [3-10]. Accompanied by a macrophage deactivation, a T-cell anergy, and an apoptotic loss of lymphoid tissues, the human clinical syndrome of CARS is characterised by uncontrolled infections followed by organ damages associated with high rates of morbidity and mortality [2].

Zinc's pro-inflammatory impact is reliably proved in various *in vitro *as well as *in vivo *models. In separated human mononuclear cells and monocytes, zinc induces the expression of cytokines [11]. In pigs, zinc upregulates the release of inflammatory mediators by the induction the heat shock response [12]. And in a rat model of endotoxemia, zinc thus reduces the rates of apoptosis and mortality [13]. During the acute phase of sepsis, this pro-inflammatory capacity can even be deleterious due to complementary properties [14]. As CARS represents an overbalanced anti-inflammatory stage of sepsis, zinc's well-known pro-inflammatory potential could be an interesting tool to recover immune capability.

To verify a rehabilitative effect of zinc on the immune system, this study analyses the impact of zinc on hemodynamics, pulmonary function, and cytokine liberation during a recurrent endotoxemia in a porcine model.

## Methods

### Animal model

The study was performed in adherence to the National Institutes of Health guidelines for the use of experimental animals observing the Interdisciplinary Principles and Guidelines for the Use of Animals in Research, Testing, and Education. Basically, we used the animal model for endotoxemia in domestic pigs as described by Klosterhalfen et al. [15]. Experiments were performed on female farm pigs (n = 10, Deutsche Landrasse) weighing 28–32 kg. The animals were randomised and divided into 2 groups (n = 5). After premedication (azaperon 3 mg/kg) and intubation (pentobarbital 6 mg/kg) anaesthesia was maintained by pentobarbital 0.1 mg/kg/min and ketamine 0.1 mg/kg/min. Arterial and venous catheters were placed in the right carotid artery and the right jugular vein. In addition, a measurement catheter of the Pulsion Cold System^® ^(PULSION Medical systems, Munich, Germany) was introduced into the femoral artery in the right groin. After the completion of all surgical manipulations, hemodynamics and respiration were stabilised over a period of 60 min. The pigs were fully anaesthetised for the whole study period of 18 hours. Fluid balance was maintained by infusion of Ringer solution (3–6 ml/kg/h). Continuous measuring of the central venous pressure served as control.

The first infusion of LPS defined hour 0 of the examination. For each application of LPS, 1.0 μg/kg E. coli endotoxin WO111:B4 (Difco Laboratories, Detroit, USA) was intravenously infused over 30 min. This LPS-infusion was repeated in each group at hours 5 and 12. To mimic an interim treatment during recurrent septicaemia, each group received an intravenous treatment (group I = saline, group II = zinc) at hour 10. As a result of former dose-finding studies [12,15], 25 mg/kg zinc-bis-(DL-hydrogenaspartate) = 5 mg/kg elementary Zn^2+ ^(Unizink^®^, Köhler Pharma, Alsbach-Hähnlein, Germany) were infused in group II. According to the suppliers instructions, the aspartate component in zinc-bis-(DL-hydrogenaspartate) has no established impact during endotoxemia. To avoid acute toxic effects, zinc as well as saline infusions were administered over 2 hours [12,15].

Hemodynamics and arterial oxygen saturation were continuously measured by arterial monitoring and pulse oxymetry (Oxyshuttle^®^, Criticaon, Hamburg, Germany). Due to former studies [12], blood samples for the measurement of respiratory parameters and cytokines (TNFα and IL-6) were registered at defined points of time (600, 615, 630, 645, 660, 720, 735, 750, 765, 780, 840, 930, 1020 min). The extravascular lung water (EVLW) and the arterial-venous intrapulmonary shunt were registered using the thermal dye dilution technique of the Pulsion Cold Z-021^® ^system (PULSION medical systems, München, Germany) [16,17]. In brief, the method uses ice-cold water and indocyanin green as indicators. Whereas cold distributes to the intra- and extravascular volumes, indocyanin green remains intravascular at 99.9%. Both indicators are injected into the right atrium, and concentration changes in time are recorded. Using the different dilution curves of both indicators, rates of the intrapulmonary shunt (Qs/Qt), the extravascular lung water (EVLW index (ml/kg) = intrathoracic thermal volume index – intrathoracic blood volume index), the cardiac output, and the systemic vessel resistance (SVRI = (systemic arterial pressure – central venous pressure) / (cardiac output / body mass)) can be calculated. The measuring points of the system corresponded with the blood gas samples.

At the end of the study period (18 hours), all animals were killed by an intravenous infusion of potassium chloride. A necropsy followed with special regard to the macroscopic findings in the lungs and the liver.

### Morphology

After removal, both lungs were weighed to indirectly measure the pulmonary endothelial leakage respectively the capillary leak syndrome. The width of the alveolar septums was defined as another indirect parameter of the interstitial edema. Therefore, multiple tissue samples from all pulmonary lobes were fixed in 10 % buffered formalin, embedded in paraffin, and sections of 4 μm were stained with hematoxylin and eosin. All measurements were quantified by morphometry at 500 different randomized locations per animal (Quantimed 500^®^, Leica Microsystems, Lübeck, Germany). In addition, the paracentral necrosis rate in the liver was quantified. After the histologic preparation of two representative samples of the right and left liver lobes, measurements were again analyzed by morphometry (Quantimed 500^®^, Leica Microsystems, Lübeck, Germany).

### Enzyme-linked immunosorbent assay (ELISA) of TNFα and IL-6

The plasma concentrations of TNFα and IL-6 were measured by cross-reactive (human) ELISA kits according to the supplier's instructions (Biozol, Hamburg, Germany). All procedures were sandwich ELISAs. The molecules of interest were first bound by immobilized primary monoclonal antibodies, afterwards washed free and finally bound to polyclonal antibodies. The visualization followed by production of color after a peroxidase reaction. The color intensity was proportional to the amount of bound conjugation and thus, to the amount of present cytokine. The absorbance was measured using a Tito-Tek-Multiscan MK" ELISA reader (Flow ICN, Meckenheim, Germany) at 490 or 450 nm comparing the samples with pooled plasma from controls with increasing amounts of recombinant cytokine. The pooled plasma did not contain detectable concentrations of endogenous cytokines.

### Statistics

For statistical analysis, the Statistical Package for Social Sciences (SPSS^®^) software was used. The significance of the pulmonary, hemodynamical, and biochemical parameters was tested by a corrected variance analysis. In case of significant differences, an independent *t *test followed. P-values < 0.05 were considered significant. All data was expressed as mean ± standard deviation (SD).

## Results

### Respiratory parameters

**Figure 1:**
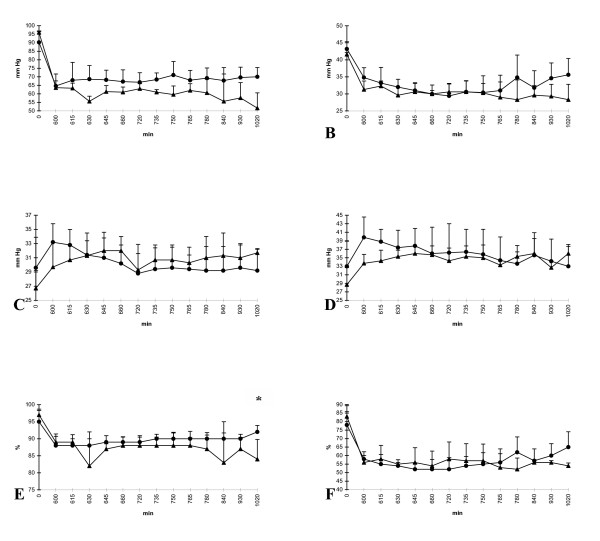
**A-F **Respiratory parameters: (A) arterial oxygen pressure, (B) venous oxygen pressure, (C) arterial carbon dioxide pressure, (D) venous carbon dioxide pressure, (E) arterial oxygen saturation, and (F) venous oxygen saturation; group I (saline) = triangle and group II (zinc) = circle; *p < 0,05; x-axis nonlinear

After infusion of zinc (t = 600–720 min), the pigs of group II merely trended to an only partly improved pulmonary function compared to group I (saline) (Fig 1A–F). The arterial oxygen pressure was continuously measured higher reaching significance only at two times (t = 630 min and t = 1020 min). The venous oxygen pressure and the arterial oxygen saturation continuously showed higher values in the zinc-group, too. However, only once, the arterial oxygen saturation reached significance (t = 1020 min). The venous oxygen saturation and the arterial and venous carbon dioxide pressure demonstrated no significant differences. After the last infusion of LPS (t = 720 min), group II (zinc) showed steady measurements. In contrast, group I (saline) demonstrated a further slight decrease of the arterial oxygen pressure and the arterial oxygen saturation.

### Extravascular lung water (EVLW) and intrapulmonary shunt

**Figure 2:**
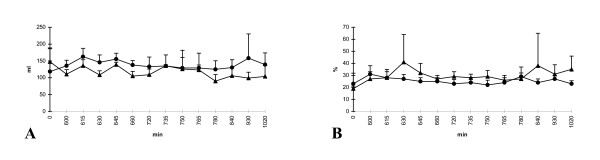
**A-B **Course of (A) extravascular lung water (EVLW, ml/kg body weight) and (B) intrapulmonary shunt (Qs/Qt, %); group I (saline) = triangle and group II (zinc) = circle; *p < 0.05; x-axis nonlinear

Analysing the thermal dye dilution data, EVLW and intrapulmonary shunt revealed no significant differences in both groups. In detail, the results were controversial. Group II (zinc) tended to result in higher rates of EVLW compared to group I (saline). In contrast, the intrapulmonary shunt consistently showed lower measurements (Fig 2A–B). However, all differences were not significant.

### Hemodynamics

**Figure 3:**
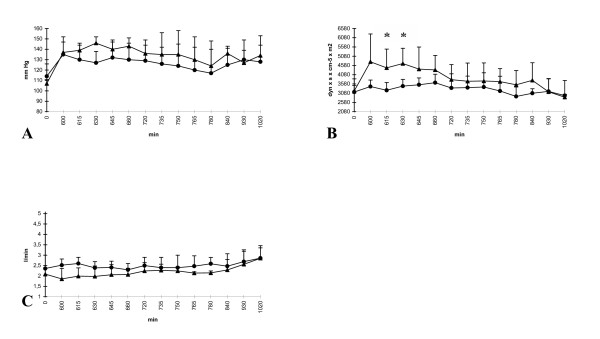
**A-C **Hemodynamics: (A) mean systemic arterial pressure (SAP), (B) systemic vessel resistance index (SVRI), (C) cardiac output; group I (saline) = triangle and group II (zinc) = circle; *p < 0.05; x-axis nonlinear

In general, the hemodynamics revealed no impressive differences. Compared to group I (saline), the mean systemic arterial blood pressure (SAP) slightly decreased in group II during the infusion of zinc. Later on, both groups showed almost similar levels without significant differences (Fig 3A). Though zinc obviously induced no gageable effect, the systemic vessel resistance index (SVRI) initially showed lower measurements in group II (zinc). Compared to group I (saline), the difference reached significance at two times (615 and 630 min). Induced by a decline of SVRI in the saline group, both groups finally showed a parallel course (Fig. 3B). Measurements of the cardiac output revealed no remarkable differences at any time (Fig. 3C). Regarding all parameters, the last application of LPS (t = 720 min) induced no further changes in both groups.

### Inflammatory mediators TNFα and IL-6

**Figure 4:**
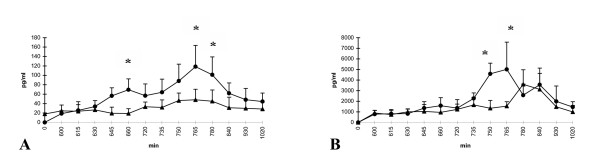
**A, B **Course of the cytokines TNFα (A) und IL-6 (B); group I (saline) = triangle and group II (zinc) = circle; *p < 0.05; x-axis nonlinear

After zinc-infusion, TNFα in group II steadily increased compared to group I (saline). Boosted by the last LPS-infusion, this effect even reached significance but persisted only temporary. At the end, both groups showed almost similar results. In group I (saline), the level of TNFα showed no remarkable changes (Fig. 4A).

The expression of IL-6 was not influenced by zinc. However, the last application of LPS induced a drastic increase of IL-6 in group II (zinc). In parallel to TNFα, this increase temporary reached significance compared to group I (saline). Later on, IL-6 increased to a smaller amount in group I (saline) as well. Caused by a parallel decrease in group II (zinc), both groups finally revealed similar results (Fig. 4B).

The significant increases of TNFα and IL-6 did not correlate with the measured differences in hemodynamics or pulmonary parameters.

### Morphology

With an increased alveolar width (group I: 11.3 μm, group II: 12.2 μm) and an elevated rate of the paracentral liver necrosis (group I: 4.3 %, group II: 7.9 %), both groups showed the typical signs of a septic organ damage. Both measurements revealed a broad range and the differences were not significant. The weight of the lungs (group I: 320.8 g, group II: 338.6 g) was measured higher in group II (zinc). Again, the results included high standard errors and did not reach significance.

## Discussion

Administered as a prophylaxis, zinc's pro-inflammatory impact protects from septic cellular damages *in vivo *as well as *in vitro *[11-13,18-20]. In a porcine model of acute endotoxemia, this pro-inflammatory effect even leads to deleterious results [14]. Thus, zinc could be predestined to restore the depressed immune capability during CARS. To our knowledge, the application of zinc during a recurrent endotoxemia *in vivo *is not yet described in literature.

To mimic a prolonged and repeated endotoxemia comparable to the human CARS-syndrome, we used the porcine model introduced by Klosterhalfen et al. [15]. The chronic form of endotoxemia was induced by recurrent LPS-infusions. In contrast to high dosage or bolus regimens, this application form more reliably imitates the clinical course of human sepsis [15]. Firstly, the typical septic organ damages found during necropsy confirmed the usability of this model. In both groups, the alveolar width and the rate of the paracentral liver necrosis were increased. Nevertheless, we found no significant differences comparing both study groups. Obviously, the application of zinc did not protect from cellular damages in our setting. Due to former dose finding studies in recurrent endotoxemia, this should rather not be a question of dosage [12,15]. However, kinetics in recurrent and acute endotoxemia are different [12,14,15,18], and zinc's optimal application rate still needs to be defined. In our opinion, the lack of impact more likely could derive from a too short registration time. Possibly, observations limited to 18 hours are insufficient to draw conclusions about the late state of LPS-induced endotoxemia.

In general, our data confirm, that the application of zinc in between a recurrent endotoxemia is well tolerated. With an almost parallel course, hemodynamic changes were rare in both groups. Besides a slight and temporary decrease of the systemic arterial pressure in the zinc-group, we did not find a relevant hemodynamic depression neither induced by zinc nor by LPS. These results contrast the deleterious pro-inflammatory impact of zinc during the acute phase of sepsis *in-vivo*, where complementing effects severely degraded hemodynamics and lead to an early death after 3 hours [14]. However, we observed no positive effect on the hemodynamic variables as well. Thus, the application of zinc in our model of recurrent endotoxemia *in vivo *is feasible but without measurable beneficial effects in hemodynamics.

The respiratory parameters reproduced the hemodynamic findings, as remarkable differences in both groups were very rare. Again, the application of zinc was well tolerated. In detail, the animals of group II (zinc) even tended to a slightly improved pulmonary function. However, significant differences of clinical relevance were missing. In our setting, zinc apparently not achieved its protective effect on pulmonary function and cell integrity reported on various studies [18-20]. Besides a longer observation time, this could be clarified by measuring the heat shock response (HSR) representing a possible pathway [13,18,20-23]. Regarding extravascular lung water (EVLW) and intrapulmonary shunt, our results were even conflicting. In conclusion, they could prove neither any protective nor any negative impact associated to zinc. Referring to their prognostic value, these parameters currently rank even higher than simple reflections of the gas exchange [24]. Thus, our findings on this field underline the lack of a clinically relevant pulmonary protection supposed above.

Regarding the liberation of the cytokines, our results remain disappointing. The changes of TNFα and IL-6 after the last LPS-application in the zinc-group revealed an increased liberation of cytokines, but the effect remained short-termed. Significant differences were only sporadically found. Except for a questionable temporary boosting, this phenomenon cannot represent a reliable reactivation of the pro-inflammatory response. Obviously, the inductive potential of zinc on the expression of pro-inflammatory cytokines [11,12,14,19,25] has no long-lasting impact in our model of recurrent endotoxemia. As the infusion of LPS in the saline-group additionally induced no measurable decrease in cytokine liberation, this may be again a question of observation time.

## Conclusion

In conclusion, the application of zinc in our experimental setting of recurrent endotoxemia is feasible and induces no harmful effects. However, we could not derive any protective or rehabilitative effect of clinical relevance on respiration, hemodynamics, or cytokine liberation. Though the rate of septic cellular damages in this animal setting is quite similar to those seen in patients with chronic sepsis, it cannot completely reflect the situation in humans. Time course, dosages, and perhaps even the LPS-model must be challenged [26]. As multiple *in vitro *and *in vivo *studies as well as clinical trials point out that depressed plasma levels of pro-inflammatory cytokines are accompanied by high rates of lethality [6-8,27-30], the restoration of the immune capability remains a challenging therapeutic aim during prolonged endotoxemia. Despite our deflating results, further pre-clinical studies including long-term observations and measurements of the heat shock response have to be established to define zinc's role in this context.

## Competing interests

The author(s) declare that they have no competing interests.

## Authors' contributions

C.J. Krones conceived of the study and design, carried out measurements, co-ordination, draft manuscript

B. Klosterhalfen conceived of the study, carried out measurements, co-ordination, reviewed manuscript

M. Anurov participated in study design, carried out measurements, co-ordination

M. Stumpf participated in study design, reviewed results and manuscript

U. Klinge conceived of the study, reviewed manuscript

A. Oettinger conceived of the study, participated in study design, reviewed results and manuscript

V. Schumpelick participated in study design, reviewed manuscript

## Pre-publication history

The pre-publication history for this paper can be accessed here:


